# Oxford-Based Clinically Relevant Interview Skills in Psychiatry Course (Ox-CRISP)

**DOI:** 10.1192/bjo.2024.277

**Published:** 2024-08-01

**Authors:** Fatima Ahmed, Shah Tarfarosh, Katherine Reid, Heidi Cooper

**Affiliations:** Oxford Health NHS Foundation Trust, Oxford, United Kingdom

## Abstract

**Aims:**

Ox-CRISP is an innovative trainee-led course using Near-Peer teaching, designed to empower new junior doctors in psychiatry to enhance their diagnostic and communication skills. The course provides patient centered simulation scenarios around interview skills in Psychiatry aiming to improve patient safety, boost confidence of junior doctors and promote effective clinical strategies. The curriculum covers a wide range of topics, including mood disorders, psychotic disorders, anxiety disorders, personality disorder, intellectual disability including CAMHS, old age psychiatry, and substance use disorders.

**Methods:**

- Curriculum Content: Covers 9 prevalent psychiatric scenarios.
- Teaching Strategy: Evidence-based, co-produced with experts by experience.
- Teaching Approach: Near-peer teaching for safe, supportive learning.
- Assessment: Pre- and post-course questionnaires to track learner progress.
- Implementation: Offered to junior doctors in Psychiatry at Oxford Health NHS Foundation Trust's Medical Education department.

**Results:**

The qualitative data showed that the course, run through multiple sessions, resulted in improved confidence & competence of trainees in clinical practice, positive impact on trainers & trainees, and enhanced well-being of junior doctors. The cope of impact affected patient care across two counties in South-East England.

**Conclusion:**

To our knowledge, this is a unique course of its kind in the UK. The OxCRISP course represents an innovative and effective approach to mental health education that provides learners with the skills and knowledge they need to provide high-quality patient care. The success of the OxCRISP course demonstrates the value of a patient-centred approach to medical education and highlights the importance of ongoing training and mentorship for mental health professionals.

